# Elements of the Principles and Practice of Midwifery

**Published:** 1848-05

**Authors:** 


*Elements of the Principles and Practice of Midwifery.* By David H. Tucker, M. D., Professor, etc., with numerous illustrations. Philadelphia: Lindsay and Blackiston. 8vo. pp. 405.

We have received the Principles and Practice of Midwifery, by David H. Tucker, M. D.; being the first number of the Medical Practitioners and Students' Library.

We have hastily examined this volume, and think it combines the merits of several of the larger works on obstetric science, in a very condensed and economical form. There have been several works of a similar character published in Europe and America within the last few years, but this compendium commends itself to the consideration of the profession by reason of its conciseness, and from that fact that it is an American production, and brings the student down to the present moment in all the improvements in the science.

In relation to the theory of generation, he adopts the theory of the periodic expulsion of ova, substantially, as taught by M. M. Bischoff, Raciborski and Pouchet.

In the mechanism of labour, Dr. Tucker has very judiciously (as we think) simplified and diminished the number of positions in his classification, making but four instead of six or eight, as described by some modern writers. We might, doubtless, in the early stage of labour, find the vertex pointing to almost any part of the pelvic brim; but so soon as the head engages in the superior strait, it would seem that the long diameter of the fœtal head must occupy the oblique or long pelvic diameter. Indeed we believe this will always hold true, when the relative portions of the head and pelvis are so nearly the same as to make it a matter of importance what relations the former holds to the latter. In cases of pelvic deformity, the head will accommodate itself to the peculiar mal-formation present, and will be an exception to any rule which may be laid down as applicable to the normal pelvis. We would therefore adopt the views of Neagle, and can perceive no propriety in multiplying the number of positions as Ramsbotham and Mad. Le Chapell have done.

We think Dr. T. too much inclined to favor the innovation of Dr. Simpson in the treatment of placenta prævia. In the main, however, we can concur with Dr. T. in his views of practice, and are happy to see that his work embraces all the improvements of modern practice in surgical midwifery; and, so far as we are aware, furnishes the most complete and systematic work, for its price, extant. The illustrations are appropriate and well executed, and, from the limited examination which we have been able to bestow upon the work, we are induced to regret only, that Dr. Tucker, who shows himself fully competent for the task, by his judicious discrimination in this undertaking, did not extend his labors, and provide for the profession what, we consider a great *desideratum*, a *cyclopædia of obstetric medicine*. In short, perform for the practitioner of midwifery (by collecting in one extensive work, everything which relates to the female and her diseases,) what Tweedie, or Copland, or Forbes have accomplished for the general practitioner. J. P. W.

